# Health Professionals' Perspectives on the Efficacy of Using Comprehensive Care to Improve Outcomes in Patients With Traumatic Injury

**DOI:** 10.1097/jnr.0000000000000396

**Published:** 2020-06-29

**Authors:** Hsing-Ju LU, Hsiu-Mei HUANG, Tsai-Yun HSIAO, Chang-Chiao HUNG, Wei-Ting LIN, Bih-O LEE

**Affiliations:** 1 MSN, RN, Supervisor, Department of Nursing, Chiayi Chang Gung Memorial Hospital, Taiwan, ROC; 2 MSN, RN, Supervisor, Tzu-Yuan Hospital, Taiwan, ROC; 3 MSN, RN, Lecturer, Chung-Jen Junior College of Nursing, Health Sciences and Management, Taiwan, ROC; 4 PhD, RN, Associate Professor, Department of Nursing, Chang Gung University of Science and Technology, and Associate Research Fellow, Department of Nursing, Chiayi Chang Gung Memorial Hospital, Taiwan, ROC; 5 PhD, RN, Associate Professor, College of Nursing, Kaohsiung Medical University, Taiwan, ROC; 6 PhD, RN, Professor, College of Nursing, Neuroscience Research Center, Kaohsiung Medical University, Taiwan, ROC.

**Keywords:** focus groups, traumatic injury outcomes, health professionals

## Abstract

**Background:**

Barriers related to comprehensive posttrauma care and health outcome monitoring exist. The insights and perspectives of health professionals on this issue may help integrate care experiences to provide continuous care to patients with traumatic injury.

**Purpose:**

The purpose of this study was to explore the perspectives of health professionals with regard to comprehensive care to improve the outcomes of patients with traumatic injury.

**Methods:**

Data were collected at two teaching hospitals in Taiwan. In total, 28 health professionals across various disciplines were interviewed in five focus groups.

**Results:**

Six themes were delineated, including “wound care is a primary concern for patients,” “ineffective health education during the hospital stay,” “patients and families worry about postinjury conditions,” “current continuity of care is not effective,” “lack of standards for discharge planning,” and “incorporation of interdisciplinary care to improve patient outcomes.”

**Conclusions:**

The experiences of health professionals are useful to the establishment of a foundation for trauma case management and interdisciplinary care for hospitals.

## Introduction

Survivors of traumatic injury may experience permanent disabilities that are associated with high medication and rehabilitation costs in addition to potentially tremendous homecare and nonofficial care expenses (Haagsma et al., 2016; Ministry of Health and Welfare, Taiwan, ROC, 2016). Surveys conducted in Taiwan indicate that the average age of survivors of major trauma is 45.1 years (Lee et al., 2010). The national productivity losses, demand for medical resources, and economic and emotional stresses imposed on affected families as a result of traumatic injury have become a major medical and economic burden for Taiwan (Lee et al., 2010).

Traumatic injury is generally defined as sudden-onset, physical injuries that are sufficiently severe to require systemic and immediate medical treatment (University of Florida Health, 2019). Traumatic injuries are diverse and heterogeneous in nature, reflecting the body regions involved as well as the severity of trauma (Meerding et al., 2004). Some studies have used the type of traumatic injury, such as musculoskeletal injury, as inclusion criterion for participants (Torgbenu et al., 2019). The Injury Severity Score is an international scoring system that is commonly used to evaluate and categorize traumatic injuries (Baker & O'Neill, 1976). Lee et al. (2008) conducted a study in which patients with Injury Severity Score values of 9–15 and greater than 15 were categorized as having moderate and severe traumatic injuries, respectively.

Studies exploring the outcomes of moderate-to-severe traumatic injury have found that physical function may be decreased at 3 months after injury (Lee et al., 2008), physical and functional disabilities may be present at 6 months after blunt thoracic injury (Baker et al., 2018) or musculoskeletal injuries (Torgbenu et al., 2019), and patients with multiple traumas may have difficulty with physical mobility at 12 months after injury (Dimopoulou et al., 2004). Moreover, psychosocial and cognitive disabilities have been associated with return to work (RTW) in patients with multiple traumatic injuries (Lee et al., 2018; Opsteegh et al., 2009). For example, only 40% of patients were able to work at 3 months after traumatic injury (Clay et al., 2012; Lin et al., 2013; Zieger et al., 2011). Approximately 60%–80% took more than 12 months to RTW or to find a new job. Another 20%–30% did not RTW because of disabilities (Clay et al., 2012; Lin et al., 2013).

Various studies have attempted to improve the health outcomes of patients with traumatic injury. For example, Lee et al. (2015) found that traumatic-injury-related physical symptoms and perceptions of control regarding trauma were positively affected by a nursing intervention at 3 months after injury, although the intervention did not change any other health outcomes. Vranceanu et al. (2015) conducted a study that examined “a mind body skills-based intervention” that used cognitive and behavioral treatments to help patients recover from traumatic injury over a period of 1–2 months. The findings indicate that disability and pain outcomes were changed and that the intervention affected participants' psychological and cognitive outcomes. Zatzick et al. (2013) conducted a study that involved patients who developed posttraumatic stress disorder after experiencing traumatic injury. The study used interdisciplinary case management combined with behavioral activation therapy, and the intervention significantly reduced posttraumatic stress disorder symptoms in comparison with a control group. Although previous research has accumulated substantial evidence regarding the improvement of trauma care, barriers related to comprehensive care and health outcome monitoring still exist, including the lack of trauma case management in most of Taiwan's hospitals (Lee et al., 2018) and the lack of specific discharge managers in hospitals who regularly follow up on postdischarge conditions and outcomes (Kimmel et al., 2016).

There is a lack of consensus regarding the intervention and outcome measures used for patients with traumatic injury because previous studies were conducted by a variety of different healthcare providers, including nurses (Lee et al., 2015), medical clinicians (Vranceanu et al., 2015), and psychologists (Zatzick et al., 2013). Furthermore, the timelines of past studies for intervention and follow-up of patients with traumatic injury have ranged from the acute stage (Vranceanu et al., 2015) to 12 months after injury (Zatzick et al., 2013). Therefore, integrating care across the trauma continuum may lead to improved short- and long-term health outcomes for individuals with traumatic injury. At the same time, limited research regarding the experiences of patients with traumatic injury at the acute and very early stage has been conducted. For example, only one previous qualitative study used individual interviews to assess the early recovery experiences of patients with traumatic injury (Chou et al., 2014). The findings of that study indicated that the traumatic injury itself traumatized patients. Themes from the research indicate that patients experienced acute pain and the inability to fulfill daily needs after traumatic injury and that recovery took a long time (Chou et al., 2014). Thus, appropriate interventions that meet the needs of patients with traumatic injuries are important for short- and long-term outcomes.

Clinician discussions have been used to integrate the disparate perceptions of healthcare providers to provide comprehensive care (Jelinek et al., 2014; Rowe et al., 2012). However, these studies have not typically been conducted in trauma care settings. Through clinician discussions, a number of possible improvements to continuous care have been suggested, including improvements to clinic-based interventions, assessments of patient and family needs, and implementation of related health education (Jelinek et al., 2014; Rowe et al., 2012). In this study, a comprehensive care model is defined as an integrated model that provides interdisciplinary professional practices, roles, and collaborative relationships in clinical practice (Ramont & Niedringhaus, 2012). This study used focus groups to obtain health professionals' perspectives regarding comprehensive care to improve the outcomes of patients with traumatic injury.

## Methods

This study explored the perspectives of health professionals regarding comprehensive care to improve the outcomes of patients with traumatic injury.

### Data Collection

Data were collected from November 2016 to October 2017. The researchers invited groups of health professionals from two teaching hospitals in Taiwan. These two hospitals both have 900–1,000 beds, treat 1,500–2,000 patients with traumatic injury each year, and maintain “Trauma Blue Teams.” However, neither uses an independent trauma specialist to liaise with interdisciplinary care providers and monitor postinjury patients. Patients with injury receive general surgical care in both hospitals.

Purposive sampling was used to approach potential study participants. The inclusion criteria required that participants be health professionals who had worked in surgical ward units and/or surgical intensive care units for at least 1 year. Two nursing supervisors recruited participants at each hospital based on these inclusion criteria. As suggested by Carey (1994), each focus group was composed of five to six participants. Semistructured and audiotaped interviews were adopted as the data collection method (McLafferty, 2004), and the suggestions of Doody et al. (2013) were followed in conducting the focus group discussions. The interview questions were developed based on the research purpose. The interview questions were as follows: (a) “Please talk about your experiences caring for patients with traumatic injury”; (b) “Please share some information regarding the traumatic-injury-related facilities, resources, and characteristics in your hospital”; (c) “What kinds of difficulties patients with traumatic injury or their families typically face?”; (d) “Please provide your perspective regarding comprehensive care for patients with traumatic injury”; and (e) “Please contribute some thoughts based on your medical expertise about how to refine care for patients with traumatic injury.”

In the focus group interviews, the principal investigator shared the results from two previous projects that studied nursing interventions. Each focus group began by introducing the moderator and gathering informed consent signatures, which included consent to audiotape the group discussion to facilitate analysis. A research team member moderated the focus group interviews, as she was an experienced qualitative researcher. First, the researcher introduced the interview questions, and then the participants were invited to share their perspectives and experiences based on the interview questions.

### Data Analysis

The interview content was analyzed using content analyses (Merriam & Associates, 2002). The following steps were used: (a) The transcribed recordings were read repeatedly to understand the perceptions of the participants, (b) particular sentences and any sentences that were mentioned repeatedly were marked, (c) perceptions with common features were generalized to form a theme, (d) the relationships of each theme were explored cautiously, (e) all of the themes were generalized to form the context, (f) the themes of the perceptions were formed, and (g) findings were returned to 60% of the participants to make sure the themes reflected their perceptions. Focus group interviews were discontinued when new themes ceased to emerge and after each group had been interviewed at least once.

Four standards from Lincoln et al. (1985) and Kidd and Parshall's (2000) guidance were used to achieve rigor. Credibility was established by continuous verification of the findings with the participants and researchers. The research team held discussions to ensure the credibility of the interview data. Percent agreement was applied to determine the interrater reliability between two persons based on suggestions from Feng (2014). The interrater agreement focused on the number of themes that were considered for their necessary data chunks by each independent rater (Zhao et al., 2013). Interrater agreement ranged from 78% to 84% for each theme, indicating that the level of agreement was acceptable overall. Two researchers analyzed the data independently, and then two research meetings were held to check the preliminary analysis and reach consensus with regard to the findings. Meanwhile, the participants helped clarify and confirm the emerging findings. To ensure dependability, the interviews were conducted and transcribed verbatim by the same researcher. The abundant data reflecting the participants' perceptions represented study transferability.

### Ethical Considerations

Institutional review board approval was obtained from the study hospital (Approval No. 201600766B0). The purpose of the study and the process of the interviews were explained to each participant, and written consent was obtained.

## Results

Twenty-eight health professionals were interviewed in five focus groups. Each focus group consisted of five to six participants, with 16 and 12 participants, respectively, participating from the two study hospitals. Eight participants (28.6%) were male, and 20 (71.4%) were female, including nine senior surgical nurses, five surgeons, five surgical nurse practitioners, three surgical nursing supervisors, three physical therapists, two social workers, and one trauma manager. The average job tenure and age of the participants were 15.6 (*SD* = 3.5) and 48.6 (*SD* = 10.2) years, respectively. The six themes are presented in the following sections.

### Wound Care Is a Primary Concern for Patients

Several participants said that most of their patients were concerned about the condition of their wound and wound healing. Although participants would have already provided the patients with instructions related to wound care in hospitals, patients still generally had problems with wound care. Some patients could not care for their wounds well and would call the hospital or return to a nurse station to ask for help.

One participant reported:

I feel that patients are most concerned about their wounds. Trauma patients feel anxious about the condition they are in when they get discharged from the hospital. They worry about the care they will receive after returning home. Patients are particularly worried about how the bandage on the wound should be changed at home when the stitches have yet to be removed, and about how they should seek medical attention in case of problems with the wound.

Another said:

Within the first week or two after the date of discharge, we often receive calls from patients who try to describe their wound but usually cannot do so clearly. For example, “My wound is very swollen, painful, and red and has some secretions.” Such a description makes it difficult for us to determine whether there is inflammation or whether the wound is healing. So we have to ask the patient to come see a physician. If an appointment is not possible, we will request that he or she seek emergency treatment.

### Ineffective Health Education During the Hospital Stay

The participants perceived that they generally offered sufficient health instructions to patients and their families based on postinjury conditions. The medical staff members sometimes needed to repeat their instructions several times when patients and their families could not quickly understand the content. However, patients still often failed to follow instructions after hospital discharge.

One participant stated:

I am often surprised when patients fail to listen no matter how much you tell them. You tell the patient what to pay attention to after the surgery and, the next day, the problem occurs again. Or you tell a patient not to apply too much force when walking after the surgery. The patient then goes home and has his or her family take care of him or her. The family is confused and problems occur with the wound, or the patient does not move correctly….

Another participant said:

I often think that our health instructions are clear, but, in fact, patients sometimes do not understand and many difficulties occur. Patients are informed about what to pay attention to after a fracture surgery in which metal screws are used. Also, we repeat the instructions to their family…. Trauma patients and their family members may be upset and fail to focus on care-related issues, in which case we also do not know what to do.

### Patients and Families Worry About Postinjury Conditions

The participants said that patients and their families were concerned about a variety of problems after experiencing a traumatic injury. Patients worried about physical symptoms such as pain, dizziness, and other injury complications. Other concerns focused on nursing facility arrangements and the timing of resuming work. The participants felt that patients generally had multiple problems that needed to be solved.

One participant said:

Patients worry about how to return to their previous lifestyles after the injury. We often see that family members are reluctant to tell patients that there will be no one to take care for them after they go home. Some patients want to go to special institutions (nursing homes) after being discharged from the hospital and the selection of such an institution is another major issue. Families demand that the institution must be close to home and the quality of care must be good.

Another participant said:

Most trauma patients are relatively young—20 ~ 60 years old. Patients worry about their future recovery and working ability. They are concerned about whether they can continue to work. Some patients worry about financial problems that can be caused by the injury, including medical treatment expenses and daily life expenditures. They worry about how they will cope if they cannot work in the future….

### Current Continuity of Care Is Not Effective

More than half of the participants expressed the view that they were usually very busy and needed to put aside their work to evaluate patient needs. This may place an extra burden on medical personnel. The participants understood that patients with traumatic injury have complicated conditions and that some care needs are not effectively addressed. Greater consensus among medical staff is necessary to build up continuity of care.

One participant said that:

The late implementation of rehabilitation for trauma can cause many problems and increase the number of hospital visits, wasting medical treatment resources. The time at which post-trauma rehabilitation is started is very important and can affect the patient's prognosis. However, clinicians do not have a clear understanding or consensus regarding the timing of trauma rehabilitation during the emergency treatment stage. For instance, some surgeons may prescribe rehabilitation and include this in the treatment plan, while some surgeons may believe that rehabilitation should take place after hospital discharge.

Another participant stated:

The rehabilitation period after trauma is long. The needs of trauma patients differ from those of other patients. Students, workers, and those who live alone face more problems. Patients who live alone are often unable to come to rehabilitation regularly, which results in poorer prognoses. Also, many hospitals do not provide rehabilitation services in the evenings. Insufficient rehabilitation leads to the inability to reach initial rehabilitation goals and achieve functional recovery, which causes concern among patients. The inability to fully recover makes them worry.

### Lack of Standards for Discharge Planning

Most participants stated that they usually provide health education 2–3 days before hospital discharge. Providers in the participating hospitals generally inform patients on matters requiring attention after discharge based on professional judgment. Patients usually did not have any questions during the discharge preparation period. Nonetheless, patients or family members frequently call the hospital after discharge to ask about what they should do in a particular situation. The participants suggested that hospital discharge preparation services should be standardized.

One participant said:

The difficulty lies in the fact that we do not know the best time for carrying out hospital discharge health education. Also, other than the patient, who else should be included? In many cases, family members take turns to provide care and we do not know exactly whom we need to speak to. In some cases, foreign workers provide care, and their understanding of the health education content is limited.

Another participant stated:

Patients do not actively voice their needs before going home. Often, just before their discharge, patients inform medical personnel that they do not have anyone to take care of them at home and that they do not know how their home environment should be prepared. Nowadays, each hospital has a specialist responsible for discharge preparation. However, the specialist cannot fully manage all the issues that patients have and can assist only some patients with homecare. Thus, current discharge preparation services require improvement….

### Incorporation of Interdisciplinary Care to Improve Patient Outcomes

More than two thirds of the participants reported that the care currently provided from the preoperative to postoperative stages is not comprehensive and expressed hope for improvement. Points of consensus among most of the participants included the need to set up a discharge referral unit, to integrate an interdisciplinary medical team, and to construct a systematic healthcare manual for injured patients and their families.

One participant stated:

Current health education leaflets and manuals are insufficient to prepare patients for hospital discharge and require improvements. The care provided by discharge referral centers must also be included. This is because these referral centers will evaluate patients but then not provide us (nurses) with clear updates on their situations. Subsequently, the patients will contact the related care units and the centers will conduct follow-ups. Thus, the nurses will not have a clear understanding of what is going on and what they must do.

Another participant said:

The post-trauma care that is provided may be not comprehensive. The content of a systematic healthcare manual for trauma patients should include wound care, diet, rehabilitation, psychological recovery, social resources, and insurance. Audiovisual materials could be provided to foreign caregivers and patients and family members who cannot read. These materials could then be viewed in the hospital or on a phone. It is important for interdisciplinary medical personnel to jointly discuss more comprehensive content for health education.

## Discussion

The findings of this study corresponded with the stated research purpose. The participants provided detailed information about patient and family concerns. Those concerns may not have been fully understood by health professionals in the past, and yet they are important for patients. The results of this study may reflect the fact that few studies have explored the early recovery experiences of patients with traumatic injuries, even as some have followed patients for up to 12 months after traumatic injury (Clay et al., 2012; Lee et al., 2008, 2010).

Of the various themes revealed in this study, most were associated with the complications, concerns, and difficulties perceived by patients with traumatic injuries during the early recovery stage. The participants reported facing several problems in helping patients to cope with postinjury situations. The first theme was that wound care was a primary concern for most patients with traumatic injury, suggesting that health professionals may want to focus more on different aspects of wound care, including the differences between wounds caused by accidents and those stemming from operations. Wound care for patients with traumatic injury may be either simple or complicated. Under Taiwan's National Health Insurance system, patients may be restricted to relatively short hospital stays. Standard wound care instructions tailored for wounds associated with different types of traumatic injuries may thus be important for patients and their families.

Previous research has shown that factors such as acute and chronic pain, poor physical function, cardiovascular complications, and mental problems cause postinjury disabilities (Perkins et al., 2012). However, this study found that patients and their families generally seemed not to pay attention to health education from providers. This may be because each traumatic injury was a shocking event for the patient and his or her family. Thus, the patient or family members may focus on the adjustment to the new realities of life and work before hospital discharge. In addition, most health professionals working in hospitals are very busy. Providing health education has become a routine aspect of medical work. Moreover, traumatic injuries are heterogeneous, and postinjury conditions may be complicated. Given the above factors, it may be difficult to offer effective education and instructions to prevent complications of traumatic injury. If a surgical unit does not establish systematic instructions for an injury on topics such as wound care, providers may offer relevant instructions based on their own experiences. Thus, the themes “ineffective health education during hospital stay” and “patients and families worry about postinjury conditions” also emerged in this study. These themes may result from patients and their families not having integrated postinjury care knowledge and information before hospital discharge.

The theme “current continuity of care is not effective” corresponds with the finding in Chou et al. (2014) that patients may have problems dealing with the consequences of traumatic injury. According to the theme, postinjury rehabilitation seems troublesome because therapy requires an extended amount of time to improve patient outcomes, and rehabilitation outcomes depend on the patient's condition. Meanwhile, if rehabilitation is slower than the patient expects, the patient may feel more anxious and experience other symptoms of mental and emotional distress. The best strategy may thus be to arrange a rehabilitation plan for each patient before hospital discharge. A patient with traumatic injury may either return to the hospital or go to a rehabilitation clinic for the therapy. However, one major problem is that Taiwan's National Health Insurance system does not cover rehabilitation and psychological consultation fees for all patients with traumatic injuries. Whether a patient with traumatic injury needs to consult a rehabilitation therapist or psychologist depends largely on the judgment of the patient's attending physician, but the needs of some patients may be ignored during the hospital stay, with the consequences of a traumatic injury emerging after discharge.

There was consensus among the participants regarding the first to fourth findings, which were rooted in the theme “lack of standards for discharge planning.” Furthermore, the participants perceived that the best solution for patients with traumatic injury may be encapsulated in the last theme: “incorporation of interdisciplinary care and patient education materials to improve patient outcomes.” Previous research has demonstrated a lack of consensus among health professionals regarding intervention protocols (Lee et al., 2015; Vranceanu et al., 2015; Zatzick et al., 2013). The findings of this study support the view that adopting standards for discharge preparation, interdisciplinary care, and integrated patient education materials will improve the quality of care provided to patients with traumatic injury.

Hospital care and rehabilitation programs must be based on evidence to provide the greatest benefits. Case management has been applied comprehensively to help patients with traumatic injury regain functionality in countries such as Australia (Arnold & Elder, 2013) and China (Kong et al., 2012). In addition, recently generated evidence on trauma care has touched on issues such as wound care (Virani et al., 2016), pain management (Jennings et al., 2014), postinjury rehabilitation (Faux et al., 2015), psychological and cognitive interventions (Vranceanu et al., 2015), and multidisciplinary recovery programs (Zatzick et al., 2013).

This study obtained detailed information related to provider perceptions of patient concerns and then reached consensus among the participating health professionals on establishing a standard for discharge planning using interdisciplinary care. This provides a window of opportunity to integrate all appropriate ideas into trauma care that may result in better health outcomes for patients with traumatic injury. The findings of this study may best be applied in medium-sized hospitals that do not assign a health professional such as a liaison case manager to provide postdischarge follow-up.

### Conclusions

The aim of this study was to explore the experiences of health professionals in using comprehensive care to improve outcomes for patients with traumatic injury. The findings suggest that the concerns of these patients and their families may not be fully understood. The health professionals may encourage patients and their families to participate actively in the process of care planning. Moreover, the detailed information that emerged from the health professionals' experiences may be useful in establishing an essential foundation for trauma case management and interdisciplinary care in hospitals in Taiwan and other countries.

### Implications for Practice

Interdisciplinary trauma case management should be organized and planned by hospital administrators. First, collaboration appears to have a positive impact on postinjury care. Trauma case management should establish a process for coordinating comprehensive healthcare services after a traumatic injury. Second, interdisciplinary teamwork, including efforts aimed at wound care, pain management, rehabilitation planning, and psychological interventions, should be integrated from the acute stage to an extended period of follow-up for individuals with injury. Third, interdisciplinary care may take time to succeed. Thus, surgical units should seek to establish or integrate educational and instructional materials for patients and their families. A dedicated contact window at each hospital may also be set up for patients to ask questions about postinjury care after hospital discharge. Last, as direct providers of healthcare, nurses should encourage patients to participate actively in their discharge planning.

### Limitations

This study highlighted the perceptions of health professionals regarding the care provided to patients with traumatic injury. Future studies should also assess the concerns and perceived care needs of these patients and their families. Furthermore, the focus group interviews in this study were conducted at two regional hospitals in southern Taiwan only. Thus, the findings may be specific to this region and may not be applicable throughout Taiwan.
